# Incidence of Cardiac Arrhythmias in Acute Myocardial Infarction Patients Undergoing Primary Percutaneous Coronary Intervention and Associated Outcomes During the First 24 Hours

**DOI:** 10.7759/cureus.12599

**Published:** 2021-01-10

**Authors:** Jehangir A Shah, Farah Naz, Rajesh Kumar, Muhammad Hassan, Ghazanfer Shah, Khalil Ahmed, Jamil Hussain, Khadijah Abid, Musa Karim

**Affiliations:** 1 Adult Cardiology, National Institute of Cardiovascular Diseases, Karachi, PAK; 2 Research Evaluation Unit, College of Physicians, Karachi, PAK; 3 Statistics, National Institute of Cardiovascular Diseases, Karachi, PAK

**Keywords:** acute myocardial infarction, cardiac arrhythmias, primary percutaneous coronary intervention, mortality, risk factors, outcomes

## Abstract

Background

Acute myocardial infarction (AMI) is the most life-threatening manifestation of coronary artery diseases. The majority of deaths in AMI are due to arrhythmias. Therefore, the aim of this study was to evaluate the incidence and risk factors and outcomes of cardiac arrhythmias in AMI patients undergoing primary percutaneous coronary intervention (PCI) during the first 24 hours of the index hospitalization.

Methodology

This prospective observational study was conducted at the adult cardiology department of the National Institute of Cardiovascular Diseases (NICVD), Karachi, Pakistan. Patients undergoing primary PCI were included in this study. All the patients were kept under observation for the first 24 hours of AMI and monitored through telemetry system monitoring and the incidence of cardiac arrhythmias and the outcomes were recorded.

Results

A total of 110 patients were included; the mean age was 59.6±13.1 years. Most of them were male (70.9%). Arrhythmias were observed in 89.1% of the patients, with 169 episodes. The accelerated idioventricular rhythm was the most common type of arrhythmia (37.3%) followed by sinus tachycardia (36.4%), ventricular tachycardia (22.7%), and complete heart block (20.0%). Lethal arrhythmias were observed in 64.5% (71) of the patients. During the hospital course, 65.5% developed arrhythmias during arrival to balloon time, 30% during the procedure, and 53.6% within 24 hours of the procedure. The in-hospital mortality rate was 15.5% with a significant association with the development of lethal arrhythmias within 24 hours of the procedure (21.1% vs. 5.1%; p=0.026).

Conclusions

The incidence of arrhythmias within 24 hours of hospitalization is high in patients with ST-elevation myocardial infarction (STEMI) undergoing primary PCI, and it has been observed to be associated with an increased rate of in-hospital mortality.

## Introduction

Globally, ischemic heart disease (IHD) is the leading cause of morbidity and mortality [1]. Among the atherosclerotic coronary artery diseases (CAD), the most life-threatening is acute myocardial infarction (AMI) and its associated complications. However, the majority of deaths in AMI are due to arrhythmias, which ranging from bradyarrhythmias, atrioventricular (AV) block, supraventricular tachyarrhythmias, and ventricular arrhythmias (VA) [2-3]. In addition to the arrhythmias observed in the acute phase of myocardial infarction, the reopening of an infarct-related artery may increase the risk of arrhythmias even further and serious arrhythmias may appear, which increases the mortality risk [4].

Fatal arrhythmias that can result in heart collapse and are major complications of AMI are ventricular arrhythmias (VT/VF) [5]. In patients with AMI, the bulk of VT/VF cases are within 48 hours after symptom onset. However, in the hospital period of acute coronary syndrome (ACS), the occurrence of VA has reduced, primarily due to prompt revascularization and optimal medical treatment. However, the Asian community has minimal evidence of the association between the timing of VT/VF events within 48h or > 48h after symptom onset and the prognosis of patients undergoing primary percutaneous coronary intervention (PCI) [6].

Sudden arrhythmic death, often followed by 'warning' ventricular arrhythmias, can exacerbate the acute phase of AMI [7]. The convalescent period of AMI has gained less attention while patients remain at risk of severe cardiac arrhythmias and sudden death. A significant correlation between early post-hospital mortality and the incidence of arrhythmias while in the hospital was found. However, with the trend toward even earlier discharge from hospital after AMI, the above investigation may need further reexamination [8].

The developing world has about 30% of the total yearly deaths due to cardiovascular diseases [9], which is also the leading cause of mortality worldwide. Despite considerable progress in management over recent years, CAD is the leading cause of death in Asian countries too, including Pakistan. The incidence of AMI is increasing in our population. However, reports on the incidence of cardiac arrhythmias and its risk factors among patients with AMI especially from symptom onset to hospitalization and discharge are relatively lacking in countries like Pakistan [10-11]. Prompt diagnosis and treatment determine the outcome of all the types of arrhythmias associated with AMI. This would suggest that more aggressive identification and modulation of cardiac arrhythmias in AMI patients is necessary among Asian Pakistanis. Hence, this study aims to evaluate the incidence, risk factors, and outcomes of cardiac arrhythmias in acute myocardial infarction patients undergoing primary percutaneous coronary intervention during the first 24 hours of index hospitalization at a tertiary care hospital in Karachi, Pakistan.

## Materials and methods

This was a prospective observational study conducted at the adult cardiology department of a tertiary care cardiac center in Karachi, Pakistan, from November 2019 to June 2020. A sample size of 101≈110 was estimated taking the frequency of cardiac arrhythmias as 78.7% [12] of patients with acute myocardial infarction (AMI) during the first 48 hours, confidence level = 95% and margin of error = 8%. All the patients of age 18-80 years of either gender with AMI and undergoing primary PCI were included using the non-probability consecutive sampling technique. AMI was characterized by a 12-lead electrocardiogram (ECG), such as ST-segment elevation in two or more contiguous leads or new left bundle branch block (LBBB). The threshold values for ST-segment elevation consistent with ST-segment elevation myocardial infarction (STEMI) is a J-point elevation greater than 2 mm (0.2 mV) in lead V2 and V3* and 1 mm or more in all other leads or by new or presumed LBBB. [*2.5 mm in men younger than 40 years; 1.5 mm in all women]. Typical chest pain >20 minutes (retrosternal pain with radiation to left arm or shoulder, aggravated on exertion or emotional stress, relieved with rest or nitroglycerin). Patients with preexisting arrhythmogenic substrate, previously documented history of ACS, prior cardiac or thoracic surgery, prior history of any cardiac intervention, dilated cardiomyopathy, chronic kidney disease (CKD), thyroid diseases, and patients on hemodialysis were excluded from the study.

Approval of the ethical review committee of the institution was taken prior to data collection. Prior to inclusion, the purpose and benefits of the study were explained to all participants and verbal informed consent was taken. Demographic and clinical characteristics of the patients such as age (years), weight (kg), height (cm), body mass index (BMI) (kg/m^2^), gender, obesity, smoking, diabetic mellitus, family history of CAD, and hypertension were recorded. Cardiac arrhythmias were diagnosed on the basis of ECG. All the patients were kept under observation for the first 24 hours of MI; all patients were monitored through telemetry system monitoring, and incidence of cardiac arrhythmias and outcomes were recorded. Confounding variables and bias were controlled by strictly following inclusion and exclusion criteria and stratification. Patient information was kept secured and accessible to authorized persons only.

Data were analyzed using Statistical Package for the Social Sciences (SPSS) version 23 (IBM Corp., Armonk, NY). Numeric variables were presented as mean ± standard deviation (SD) or median (interquartile range). Frequency and percentages were calculated for categorical variables. Risk factors, angiographic and procedural characteristics, and outcome (in-hospital mortality) were compared with the incidence of lethal arrhythmias within 24 hours of the procedure using the chi-square test or Fisher's exact test. Lethal arrhythmias included any arrhythmia other than accelerated idioventricular rhythm, sinus tachycardia (ST), or sinus bradycardia (SB). A P-value of ≤ 0.05 was taken as the criterion of statistical significance.

## Results

A total of 110 patients were enrolled in the study. The mean age of the patients was 59.6±13.1 years (range: 22-100 years). Most of the patients were males (70.9%). The mean BMI was estimated as 25.0±2.5 kg/m^2^ (range: 19.0-34.6 kg/m^2^). Only 10 (9.1%) patients were obese (BMI>27.5 kg/m^2^), 53.6% had hypertension, 46.4% had diabetes mellitus, 1.8% had a family history of MI, 3.6% had a family history of coronary artery disease (CAD), 32.7% were smokers, and 1.8% were alcohol consumers. All of the patients were presented with chest pain, and the median duration of chest pain was reported as 267.50 minutes with an interquartile range of 120 to 675 minutes. At the time of admission, the mean heart rate was 86.4±29.7 beats/mins and the mean serum creatinine level was 1.35±0.87 mg/dL. Most of the patients had three vessels disease (3VD) (43.6%) and the most frequent culprit vessel was the left anterior descending artery (LAD) (43.6%). Pre-procedure thrombolysis in myocardial infarction (TIMI) flow grade was 0 in most of the patients (94.5%). The clinical and demographic characteristics of the patients are presented in Table 1.

**Table 1:** Clinical and demographic characteristics TIMI = Thrombolysis in myocardial infarction

Characteristics	Total
Total (N)	110
Gender	
Male	70.9% (78)
Female	29.1% (32)
Age (years)	59.59 ± 13.1
Risk Factors	
Obesity	9.1% (10)
Diabetes mellitus	46.4% (51)
Hypertension	53.6% (59)
Smoking	32.7% (36)
Killip Class at presentation	
I	60.9% (67)
II	12.7% (14)
III	16.4% (18)
IV	10% (11)
Number of vessels involved	
Non-obstructive	0.9% (1)
Single vessel disease	29.1% (32)
Two vessels disease	26.4% (29)
Three vessels disease	43.6% (48)
Culprit coronary artery	
Diagonal	0.9% (1)
Left anterior descending artery (LAD)	43.6% (48)
Left circumflex (LCX)	17.3% (19)
Right coronary artery (RCA)	37.3% (41)
Ramus intermedius	0.9% (1)
Pre-procedure TIMI flow grade	
0	94.5% (104)
I	5.5% (6)
II	0% (0)
III	0% (0)

Arrhythmias were observed in 89.1% of the patients with 169 episodes. Accelerated idioventricular rhythm was the most common type of arrhythmias (37.3%) followed by sinus tachycardia (36.4%), ventricular tachycardia (22.7%), and complete heart block (20.0%). Lethal arrhythmias were observed in 64.5% (71) of the patients. The distribution of arrhythmias by type of myocardial infarction is presented in Table 2.

**Table 2:** Distribution of arrhythmias Inf = inferior, RV = right ventricular, Pos = posterior, Lat = lateral, Ant = anterior

Arrhythmias	Total	Type of MI						
		Inf	Inf+RV/Inf+Pos	Inf+Pot+Lat	Post	Post+Lat	Ant	Ant+Lat
Total (N)	110	24	34	1	4	2	44	1
Arrhythmias	89.1% (98)	87.5% (21)	91.2% (31)	100% (1)	50% (2)	50% (1)	93.2% (41)	100% (1)
Total episodes	169	38	49	1	7	5	68	1
Sinus tachycardia (ST)	36.4% (40)	12.5% (3)	26.5% (9)	100% (1)	50% (2)	50% (1)	54.5% (24)	0% (0)
Sinus bradycardia (SB)	5.5% (6)	12.5% (3)	5.9% (2)	0% (0)	0% (0)	0% (0)	2.3% (1)	0% (0)
Atrial fibrillation (AFib)	8.2% (9)	0% (0)	11.8% (4)	0% (0)	0% (0)	50% (1)	6.8% (3)	100% (1)
Ventricular tachycardia (VT)	22.7% (25)	20.8% (5)	11.8% (4)	0% (0)	50% (2)	50% (1)	29.5% (13)	0% (0)
Non-sustained VT (NSVT)	1.8% (2)	0% (0)	0% (0)	0% (0)	0% (0)	0% (0)	4.5% (2)	0% (0)
Ventricular fibrillation (VF)	9.1% (10)	8.3% (2)	5.9% (2)	0% (0)	25% (1)	50% (1)	9.1% (4)	0% (0)
Junctional bradycardia (JB)	1.8% (2)	0% (0)	5.9% (2)	0% (0)	0% (0)	0% (0)	0% (0)	0% (0)
Tachy-brady	1.8% (2)	0% (0)	0% (0)	0% (0)	0% (0)	0% (0)	4.5% (2)	0% (0)
Premature ventricular contractions	4.5% (5)	20.8% (5)	0% (0)	0% (0)	0% (0)	0% (0)	0% (0)	0% (0)
1st degree atrioventricular block	0.9% (1)	0% (0)	2.9% (1)	0% (0)	0% (0)	0% (0)	0% (0)	0% (0)
2nd degree Atrioventricular block	3.6% (4)	8.3% (2)	2.9% (1)	0% (0)	0% (0)	0% (0)	2.3% (1)	0% (0)
Complete heart block	20% (22)	33.3% (8)	35.3% (12)	0% (0)	0% (0)	0% (0)	4.5% (2)	0% (0)
Accelerated idioventricular rhythm	37.3% (41)	41.7% (10)	35.3% (12)	0% (0)	50% (2)	50% (1)	36.4% (16)	0% (0)

**Figure 1:**
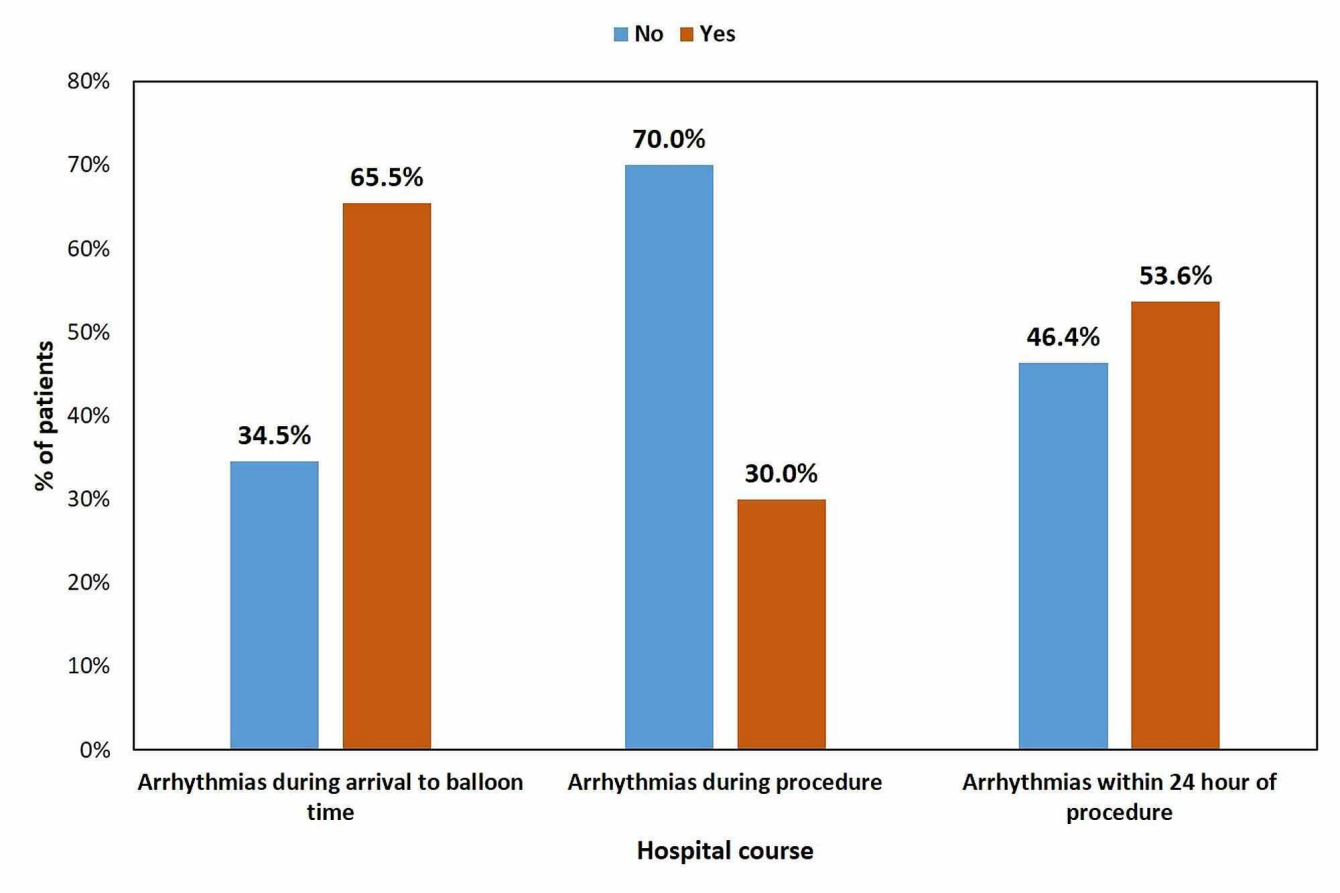
Frequency distribution of arrhythmias during the hospital course

The mean hospital stay was 3.19±2.07 days with about 31.8% of the patients with prolonged hospital stay (≥4 days). During the hospital course, 65.5% developed arrhythmias during arrival to balloon time, 30% developed during the procedure, and 53.6% developed arrhythmias within 24 hours of the procedure. The frequency distribution of arrhythmias during the hospital course is presented in Figure 1.

The frequency distribution of in-hospital outcomes stratified by the patients who developed arrhythmias within 24 hours of the procedure is presented in Table 3. During the hospital stay, mortality occurred in 15.5% of the cases, 6.4% of patients experienced bleeding events, and acute stent thrombosis occurred in 2.7% of the cases. A statistically significant association was found between mortality and the development of lethal arrhythmias within 24 hours of the procedure (p=0.026) with a mortality rate of 21.1% vs. 5.1% for patients with and without lethal arrhythmias within 24 hours of the procedure.

**Table 3:** Frequency distribution of in-hospital outcomes stratified by the development of arrhythmias within 24 hours of the procedure *significant at 5%

In-hospital outcomes	Total	Lethal arrhythmias within 24 hours		P-value
		No	Yes	
Total (N)	110	39	71	-
In-hospital mortality	15.5% (17)	5.1% (2)	21.1% (15)	0.026*
Bleeding event	6.4% (7)	7.7% (3)	5.6% (4)	0.672
Acute stent thrombosis	2.7% (3)	0% (0)	4.2% (3)	0.193
Sub-acute stent thrombosis	0.9% (1)	0% (0)	1.4% (1)	0.457

## Discussion

Acute myocardial infarction is a debilitating disease leading to multiple complications. Among them, arrhythmia is a frequently observed complication leading to death. In the present study, the mean age of patients was 59.59 ± 13.1 years old. It has been evident that older age is a risk factor for developing arrhythmias [12-13]. Male gender is more predominant as compared to females, which is analogous to two studies [14-15].

In our study, a total of 89.1% of the patients were observed to have arrhythmias along with MI, out of which 64.5% had lethal arrhythmias, which included ventricular tachycardia (22.7%), complete heart block (20.0%), ventricular fibrillation (9.1%), and atrial fibrillation (8.2%), while among non-lethal arrhythmias, accelerated idioventricular rhythm were observed in 37.3%, sinus tachycardia in 36.4%, and sinus bradycardia in 5.5% of the patients. The in-hospital mortality rate for the patients who developed lethal arrhythmias during 24 hours was significantly higher than those with non-lethal arrhythmias with a mortality rate of 21.1% vs. 5.1%; p=0.026.

Similar to our study, the overall incidence of arrhythmias after STMI is reported to be as high as 78% to 83% [12,16-18]. The conduction disturbance and arrhythmias before or during primary PCI are fairly common due to reperfusion injury and ongoing myocardial ischemia [19]. Accelerated idioventricular rhythm reported in our study is aligned with the occurrence rate of 15% to 50% reported in the literature [16-17,19]. Rate of atrial fibrillation (8.2%) and ventricular tachycardia (22.7%) are also within the range of reported incidence of 7% to 13% [18-19] and 17% to 29% [20-21]. However, the rate of complete heart block (20.0%) in our study is higher than the reported incidence of 5%-10% for a high-degree AV block [18-19]. The incidence rate of sinus tachycardia is 7% to 22% [16,18-19], which was observed to be slightly higher in our study (36.4%).

In accordance with the results of our study, Albanese M et al. observed that most of the malignant arrhythmias occurred within 96 hours of MI and found to be associated with an increased mortality rate [22].

This study has several limitations at the design stage, such as a single center-based study, small sample size, and lack of Holter monitoring. Secondly, at the execution stage, telemetry monitoring is not a routine clinical practice for all patients at our center, hence, patients who have undergone telemetry monitoring are suspected high-risk patients, which may have influenced the in-hospital mortality rate.

## Conclusions

The incidence of arrhythmias within 24 hours of hospitalization in patients with STEMI undergoing primary PCI is as high as 89.1%, and it has been observed to be associated with an increased rate of in-hospital mortality.

## References

[1] Wendelboe AM, Raskob GE (2016). Global burden of thrombosis: epidemiologic aspects. Circ Res.

[2] Taghaddosi M, Dianati M, Bidgoli JFG, Bahonaran J (2010). Delay and its related factors in seeking treatment in patients with acute myocardial infarction. ARYA Atheroscler.

[3] Mehta LS, Beckie TM, DeVon HA (2016). Acute myocardial infarction in women: a scientific statement from the American Heart Association. Circulation.

[4] Ohlow M-A, Geller JC, Richter S, Farah A, Müller S, Fuhrmann JT, Lauer B (2012). Incidence and predictors of ventricular arrhythmias after ST-segment elevation myocardial infarction. Am J Emerg Med.

[5] Masuda M, Nakatani D, Hikoso S (2016). Clinical impact of ventricular tachycardia and/or fibrillation during the acute phase of acute myocardial infarction on in-hospital and 5-year mortality rates in the percutaneous coronary intervention era. Circ J.

[6] Kalarus Z, Svendsen JH, Capodanno D (2019). Cardiac arrhythmias in the emergency settings of acute coronary syndrome and revascularization: an European Heart Rhythm Association (EHRA) consensus document, endorsed by the European Association of Percutaneous Cardiovascular Interventions (EAPCI), and European Acute Cardiovascular Care Association (ACCA). Europace.

[7] Klein HU, Meltendorf U, Reek S (2010). Bridging a temporary high risk of sudden arrhythmic death. Experience with the wearable cardioverter defibrillator (WCD). Pacing Clin Electrophysiol.

[8] Tran HV, Lessard D, Tisminetzky MS, Yarzebski J, Granillo EA, Gore JM, Goldberg R (2018). Trends in length of hospital stay and the impact on prognosis of early discharge after a first uncomplicated acute myocardial infarction. Am J Cardiol.

[9] Zuidersma M, Conradi HJ, van Melle JP, Ormel J, de Jonge P (2013). Depression treatment after myocardial infarction and long-term risk of subsequent cardiovascular events and mortality: a randomized controlled trial. J Psychosom Res.

[10] Shah BA, Khushk IA (2017). Risk factors in acute myocardial infarction patients admitted at three health centres of Sindh, Pakistan: a case control study. Khyber Med Univ J.

[11] Barolia R, Sayani AH (2017). Risk factors of cardiovascular disease and its recommendations in Pakistani context. J Pak Med Assoc.

[12] Patil PR, Khatana P, Patil DR (2017). Incidence of cardiac arrhythmias in patients with acute myocardial infarction during the first 48 hours of the onset of chest pain. Int J Adv Med.

[13] Deshpande J, Dixit JV (2008). Risk factors for acute myocardial infarction: a hospital based case control study. Health Popul Perspect Issues.

[14] Bally M, Dendukuri N, Rich B, Nadeau L, Helin-Salmivaara A, Garbe E, Brophy JM (2017). Risk of acute myocardial infarction with NSAIDs in real world use: bayesian meta-analysis of individual patient data. BMJ.

[15] Ullah I, Ali J, Faheem M, Qureshi S, Shah SFA, Khan SA, Hafizullah M (2013). Frequency of complete heart block and in-hospital mortality in patients with acute anterior wall myocardial infarction. Pak Heart J.

[16] Terkelsen CJ, Sørensen JT, Kaltoft AK, Nielsen SS, Thuesen L, Bøtker H-E, Lassen JF (2009). Prevalence and significance of accelerated idioventricular rhythm in patients with ST-elevation myocardial infarction treated with primary percutaneous coronary intervention. Am J Cardiol.

[17] Tatli E, Alicik G, Buturak A, Yilmaztepe M, Aktoz M (2013). Arrhythmias following revascularization procedures in the course of acute myocardial infarction: are they indicators of reperfusion or ongoing ischemia?. Sci World J.

[18] Bloch Thomsen PE, Jons C, Raatikainen MP (2010). Long-term recording of cardiac arrhythmias with an implantable cardiac monitor in patients with reduced ejection fraction after acute myocardial infarction: the Cardiac Arrhythmias and Risk Stratification After Acute Myocardial Infarction (CARISMA) study. Circulation.

[19] Gorenek B, Lundqvist CB, Terradellas JB (2014). Cardiac arrhythmias in acute coronary syndromes: position paper from the joint EHRA, ACCA, and EAPCI task force. Europace.

[20] Mehta RH, Califf RM, Yang Q (2007). Impact of initial heart rate and systolic blood pressure on relation of age and mortality among fibrinolytic-treated patients with acute ST-elevation myocardial infarction presenting with cardiogenic shock. Am J Cardiol.

[21] Lindholm MG, Boesgaard S, Thune JJ, Kelbaek H, Andersen HR, Kober L, Investigators D (2008). Percutaneous coronary intervention for acute MI does not prevent in‐hospital development of cardiogenic shock compared to fibrinolysis. Eur J Heart Fail.

[22] Albanese M, Alpaslan K, Ouarrak T, Merguet P, Schneider S, Schöls W (2018). In-hospital major arrhythmias, arrhythmic death and resuscitation after successful primary percutaneous intervention for acute transmural infarction: a retrospective single-centre cohort study. BMC Cardiovasc Disord.

